# Ferulated Poly(vinyl alcohol) based hydrogels^☆^

**DOI:** 10.1016/j.heliyon.2023.e22330

**Published:** 2023-11-14

**Authors:** Simone Pepi, Marco Paolino, Mario Saletti, Jacopo Venditti, Luigi Talarico, Marco Andreassi, Germano Giuliani, Gianfranco Caselli, Roberto Artusi, Andrea Cappelli, Gemma Leone, Agnese Magnani, Lucio Rovati

**Affiliations:** a Dipartimento di Biotecnologie, Chimica e Farmacia (Dipartimento di Eccellenza 2018-2022), Università Degli Studi di Siena, Via A. Moro 2, 53100, Siena, Italy; b Rottapharm Biotech, Via Valosa di Sopra 7, 20052, Monza, Italy; c National Interuniversity Consortium of Materials Science and Technology (INSTM), Via G. Giusti 9, 50121, Firenze, Italy

**Keywords:** poly(vinyl alcohol) (PVA), Ferulic acid, Hydrogel, Rheology, Wound dressing

## Abstract

New graft copolymers were prepared by reaction of poly (vinyl alcohol) (PVA) with mono-imidazolide or bis-imidazolide derivatives of ferulic acid (FA) with the formation of ester bonds. The obtained graft copolymers, thanks to the crosslinking capability of FA, formed in water strong gels as verified by rheological analyses. The resulting hydrogels were characterized to evaluate their applicability as wound dressing. In this perspective, their capability to absorb and retain a large amount of fluid without dissolving was verified by swelling kinetics and Moisture Vapour Transmission Rate measurements. Their stability towards mechanical solicitations was assessed by quantifying elasticity, compliance, stress-relaxation, and adhesivity properties. The analyses pointed out that hydrogel PVA-FA2-3 obtained by feruloylation of PVA with bis-imidazole derivative of ferulic acid using an acylation agent/polymer molar ratio 0.03/1 resulted the best candidate for the foreseen application.

## 1 Introduction

Skin protects the body from external attacks of pathogens and chemicals [1] and its functional and structural integrity is restored via a series of complex cellular and biochemical processes called wound healing [2]. Improper treatment of wounds can have serious consequences [3], so gauzes, foams, hydrocolloids, and nanoparticles have all been developed [4] and tested to find the best preparation to accelerate wound healing. Since an ideal wound dressing should be able to absorb abundant exudates, maintain a moist, cool and clean environment and guarantee an adequate oxygen exchange and water permeability, hydrogels can be seen as the most appropriate candidates for this purpose [5]. Indeed, hydrogels can absorb wound exudates, retain a large amount of water, keep the wound environment moist, allow the effective delivery of nutrients and oxygen, simulating extracellular matrix and thus providing a microenvironment suitable for wound healing [[6], [7], [8]]. Different polymers have been studied to realize hydrogel wound dressings, such as cellulose, silk fibroin, alginate, gelatine, and chitosan [9]. In the last decades, several studies highlighted the eligibility of polyvinyl alcohol (PVA) as wound dressing material [[10], [11], [12], [13]]. PVA, a water-soluble nontoxic and biocompatible polymer, is approved by US Food and Drug Administration (FDA) and European Medicine Agency (EMA) for medical and pharmaceutical applications [14]. PVA-based crystalline physical hydrogels can be easily obtained by freeze-thawing method, however a high degree of crystallinity could be a limiting factor for the foreseen application since wound dressing requires an adequate stretchability. To reduce the crystallinity degree of PVA hydrogels, the introduction of novel functional groups through polymer modification represents a promising approach [15]. In this perspective, we functionalized PVA by inserting ferulic acid (FA) moieties to reduce its crystallinity. Ferulic acid (FA, 4-hydroxy-3-methoxycinnamic acid) was chosen to take advantage of both its well-known pharmacologic actions (i.e. anti-inflammatory, anti-bacterial, anti-aging, and sunscreen features properties [[16], [17], [18], [19], [20], [21]]) and physical crosslinking activity. Indeed, FA, synthesized by phenylpropanoid pathway, is involved in the cross-linking of polysaccharides and proteins during cell wall synthesis thanks to an ester linkage between FA carboxylic group and the primary alcohol group of arabinose side chains of arabinoxylans. The chemical modification of PVA with FA resulted in the formation of PVA-FA graft copolymers. These copolymers were designed to combine the outstanding film-forming and adhesive characteristics of PVA with the ability of ferulate residues to establish interchain hydrogen bonds, leading to the formation of physically cross-linked and strong gels upon exposure to water. Moreover, due to the absence of free aminic and carboxylic groups (differently from other tested polymers, such as a hyaluronan, alginate, etc.) PVA-FA hydrogels could have a lower sensitivity to environmental pH changes thus avoiding the consequent shrinking/swelling that could alter wound protection. To the best of our knowledge, this is the first study investigating the ability of ferulic acid functionalized PVA physical strong gels as wound dressing. The work described herein was finalized to evaluate the potential applicability of PVA-FA graft copolymers in wound healing. The properties of an optimal wound dressing, i.e. swelling capability, moisture vapour transmission and adhesivity, were quantified by thermal and rheological measurements.

## 2 Experimentals

### 2.1 Synthesis of PVA-FA graft copolymers

(E)-3-(4-Hydroxy-3-methoxyphenyl)-1-(1H-imidazole-1-yl)prop-2-en-1-one (AA1) and (E)-4-(3-(1H-Imidazole-1-yl)-3-oxoprop-1-enyl)-2-methoxyphenyl-1H-imidazole-1 carboxylate (AA2) were prepared from ferulic acid (FA, Sigma Aldrich) and one or two equivalents of 1,1′-carbonyldiimidazole (CDI, Sigma Aldrich) respectively [22]. PVA-FA graft copolymers were prepared modifying the procedures used to differently functionalize Hyaluronic acid and previously reported [[23], [24], [25], [26], [27]]. Briefly, PVA (Merck Emprove 28–99) was dissolved in formamide (50 mL/1 g PVA) at about 130 °C and the resulting clear solution was cooled to room temperature. Triethylamine (1 equivalent) and the appropriate amount of freshly prepared mono-imidazolide (AA1) or bis-imidazolide (AA2) (as finely powdered solids) were sequentially added. After stirring the yellow-orange reaction mixture for 16 h at room temperature, the polymer was precipitated by treatment with acetone, collected by filtration, purified by washing with acetone for three times, and finally dried under reduced pressure to afford the expected PVA-FA derivatives. Grafting reaction was checked by FTIR and NMR.

^1^H NMR spectra were recorded with a Bruker DRX-400 AVANCE spectrometer in DMSO‑*d*_6_ (TMS as internal standard): the values of the chemical shifts are expressed in ppm and the coupling constants (J) in Hz. All the spectra were processed using MestReNova software with an Apple MacBook Pro computer.

FTIR spectra of lyophilized PVA-FA samples were recorded between 4000 cm^−1^ and 750 cm^−1^ using a Nicolet Thermo 5700 apparatus (resolution: 2.0 cm^−1^). Baseline and spectra correction were performed using OMNIC correction ATR software [28].

### 2.2 Characterization of PVA-FA graft copolymers

#### 2.2.1 Solubility test

The solubility of PVA-FA derivatives was tested in three different solvents. Briefly, 10 mg of each graft copolymer sample were weighted into a vial and the suitable amount of the solvent (i. e. 0.85 g of DMSO‑*d*_6_, 1.0 mL of D_2_O, or 1.0 mL of D_2_O containing 1.0 mg of sodium carbonate) were added to each vial and the resulting mixture was stirred at room temperature for 24 h.

#### 2.2.2 Swelling ratio

The swelling ratio (SR%) was calculated using the following equation (Equation 1):

SR%=[(Ws–Wd)/Ws]*100

where Ws and Wd are the weight of swollen and dry sample, respectively. Ws was obtained by dipping samples (15 mg) in bi-distilled water in a thermostatic bath at 37 °C. The weight was monitored until it reached the plateau.

#### 2.2.3 Thermogravimetric analysis

Q600 analyzer (TA Instruments-Waters, USA) was used to quantify samples weight loss as a function of heating. Dried samples (10–15 mg) were put in a platinum crucible and heated from 30 °C to 600 °C (heating ramp 10 °C/min) under nitrogen gas. Swollen samples (20 mg in weight) were heated up to 200 °C (heating ramp 10 °C/min) under nitrogen gas, to quantify their total water (WH) [29]. Data elaboration was performed using TA Instruments Universal Analysis 2000 (v. 4.5.4) software.

#### 2.2.4 Differential scanning calorimetry (DSC)

A differential scanning calorimeter (DSC-TA Q2000) was used to measure the crystallinity degree of all the samples and the ice melting behaviour of swollen samples. For the crystallinity degree the following procedure was used: 5–10 mg of dry **PVA-FA** polymers were sealed in aluminium pans and heated from room temperature to 270 °C, with a rate of 10 °C/min. Swollen samples, 15–20 mg in weight, were inserted in aluminium pans, cooled to −40 °C and then heated to 40 °C (heating rate 2 °C/min) to measure the ice melting enthalpy [29]. Data elaboration was performed using TA Instruments Universal Analysis 2000 (v. 4.5.4) software.

#### 2.2.5 Moisture vapour transmission rate (MVTR)

The moisture vapour transmission rate was determined following the procedure reported by Karthick et al. [2]. Briefly, swollen samples were placed onto a co-colture filter at the top of a 50 mL centrifuge tube filled with water (37.5 mL). Then, the centrifuge tubes were maintained at 37 °C and 65 % of RH (relative humidity). At regular time interval, the weight of the centrifuge tube was measured to determine the weight loss. An uncovered centrifuge tube was used as control sample. The weight loss was plotted against time and the following formula (Eq. 2) used to calculate MVTR,

$$\text{MVTR}=\left[\left(\text{Slope}*24\right)/A\right]g/m^{2}/\text{day}$$

where, A represents the scaffold area in m^2^.

#### 2.2.6 Rheology

Rheological analysis of all samples was conducted using a rheometer (DHR-2 TA Instruments, New Castle, DE, USA) equipped with a plate-plate geometry (Ø = 4 cm). All the experiments were conducted at 37 °C. Preliminary strain sweep tests were run to identify the Linear Viscoelasticity Region (LVR) recording elastic modulus of the materials at fixed oscillation frequencies (i.e., 0.1 Hz, 1 Hz and 15 Hz) while varying the strain % from 0.1 to 10 %.

Elastic and viscous moduli in shear and in compression mode (G' -G″ and E′- E″, respectively) of samples (shear strain: 0.25 %) were measured as a function of oscillation frequency in the range 0.01–15 Hz.

Stress-relaxation experiments were carried out to assess the relaxation behaviour of samples following continuous exposure to a fixed strain. Specifically, strains of 5 %, 20 %, 30 %, and 100 % were applied, and the hydrogels were allowed to relax for 600 s.

Creep-recovery test was conducted maintaining the sample at a constant stress of 50 Pa for 1800 s and then monitoring the recovering percentage. Data elaboration was performed using TA Instruments TRIOS version 4.1.1.33073.

#### 2.2.7 Adhesion test

The adhesion strength of the samples was quantified following the methodology reported by Bakthawara et al. [30]. Briefly, samples were removed with a pull-off speed of 0.1 mm/s. The peak normal force was plotted against time and the area under the curve was calculated to obtain the adhesion strength.

### 2.3 Statistical analysis

Multiple comparisons were conducted using a one-way ANOVA, and individual variations were assessed through post-hoc Tukey's test following the confirmation of significant intergroup distinctions by ANOVA. Variations with a p-value less than 0.05were considered statistically significant.

## 3 Results and discussion

### 3.1 Synthesis of PVA-FA graft copolymers

**Scheme 1:**
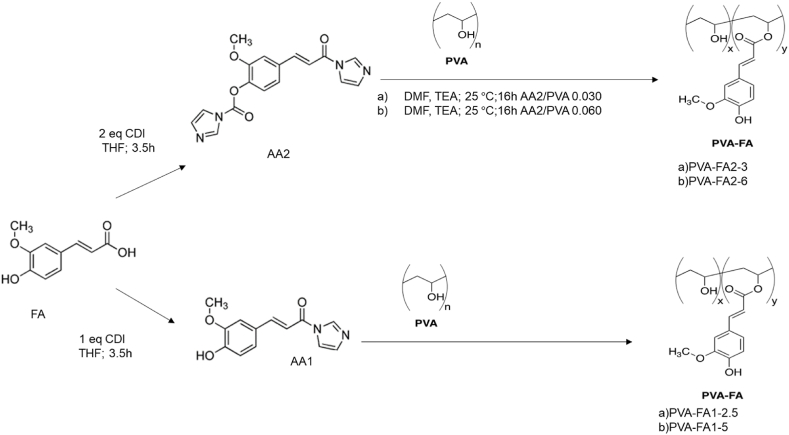
Esterification of PVA by using FA N-acylimidazole derivatives 1 or 2.

PVA-FA graft copolymers were prepared as depicted in Scheme 1.

FA was activated with 1 or 2 equivalents of 1,1′-carbonyldiimidazole (CDI) to obtain two reactive intermediates, namely mono-imidazolide [i.e. (E)-3-(4-hydroxy- 3-methoxyphenyl)-1-(1H-imidazole-1-yl)prop-2-en-1-one] (AA1) and bis-imidazolide [i.e. (E)-4-(3-(1H-imidazole- 1-yl)-3-oxoprop-1-enyl)-2-methoxyphenyl 1H-imidazole-1-carboxylate] (AA2). Both FA derivatives have been found to be effective reagents in the feruloylation of PVA to give PVA-FA graft copolymers. This is achieved througha reaction between the hydroxyl groups of PVA and the mono-imidazolide or bis-imidazolide activated carboxylic groups of ferulic acid, leading to the formation of an ester bond.

The stoichiometric ratio between the acylating agent (AA, 1 or 2) and PVA (AA/PVA ratio) was varied from 2.5 % to 5 % molar in the case of the acylation with mono-imidazolide 1, and from 3 % to 6 % in the case of the acylation with bis-imidazolide 2 (Table 1).

**Table 1:** Reaction parameters used for the functionalization of PVA polymer.

Copolymer	PVA (g)	PVA (mmol)	AA	AA (mmol)	AA/PVA Ratio (%)
PVA-FA1-2.5	0.25	5.68	1	0.143	2.5
PVA-FA1-5	0.25	5.68	1	0.286	5
PVA-FA2-3	6.5	148	2	4.43	3
PVA-FA2-6	6.5	148	2	8.87	6

**Fig. 1:**
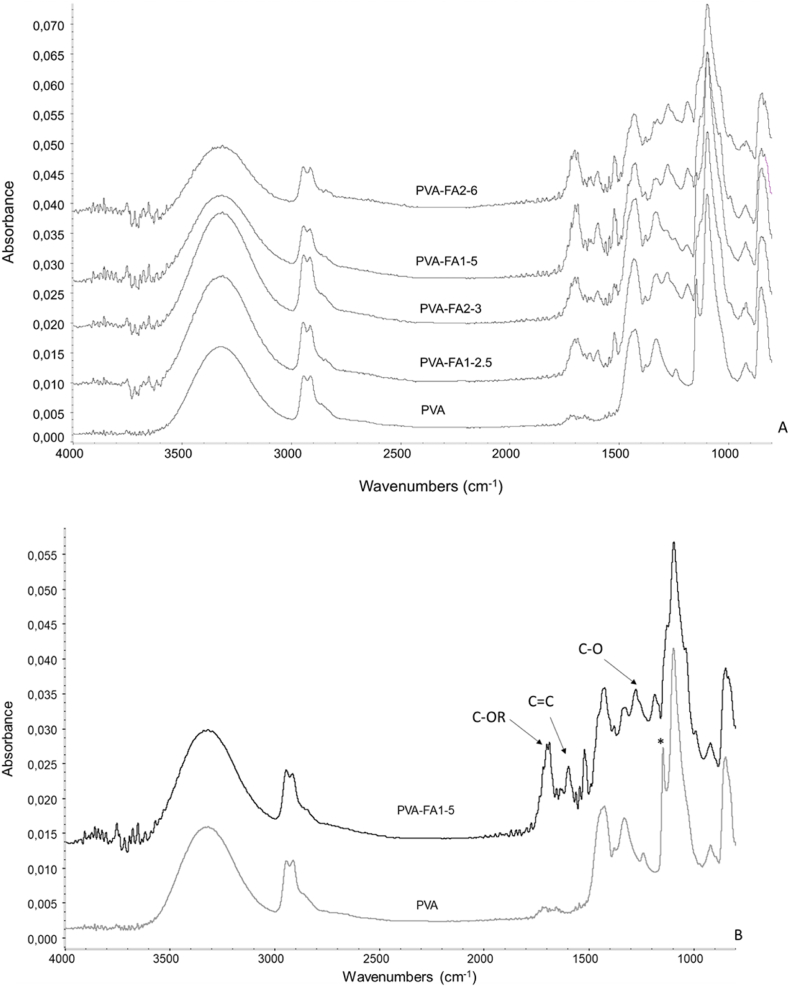
**A:** IR spectra of native PVA polymer and PVA-FA copolymers at dry state; B: Comparison between IR spectra of native PVA and PVA-FA1-5 (*: crystallinity band 1142 cm^−1^ [31]).

ATR-IR spectra of native and functionalized PVA samples were recorded to confirm the acylation reaction (Fig. 1a,b).

**Fig. 2:**
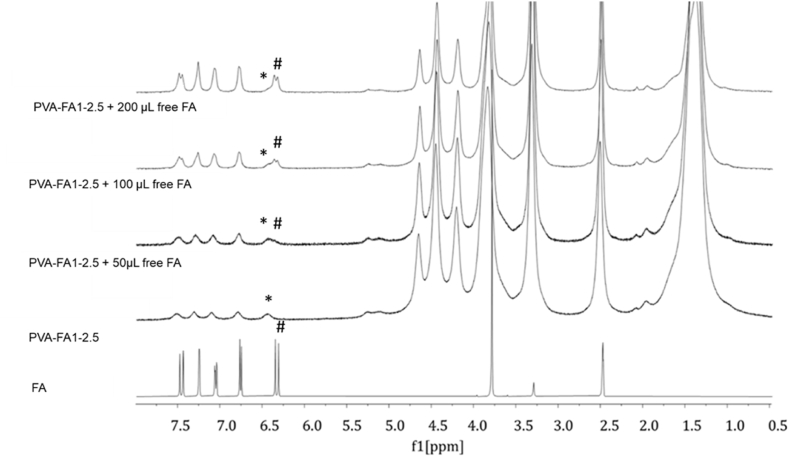
Comparison of ^1^H NMR spectra obtained with PVA-FA1-2.5 derivative (DMSO‑*d*_6_, 400 MHz) in the absence (trace 2 from the bottom), or in the presence of increasing amounts (traces 3–5 from the bottom) of free FA (added as a solution in DMSO‑*d*_6_) with that obtained with pure FA (bottom trace).

All PVA-FA graft copolymers showed superimposable IR spectra as shown in Fig. 1A. In Fig. 1B IR spectra of native PVA and PVA-FA1-5 were compared to highlight the main differences due to the reaction between OH groups of PVA and the mono-imidazolide and bis-imidazolide activated carboxylic groups of ferulic acid. Both spectra showed the typical bands of the polymeric component, i.e. the band at 3328 cm^−1^ due to intra and inter molecular H-bonded OH stretching, the double band at 2913-2850 cm^−1^ related to CH_2_ asymmetric and symmetric stretching, the band centred at 1439 cm^−1^ due to CH_2_ bending and the band at 1098 cm^−1^ due to C–O and alcoholic OH bending. The effectiveness of FA grafting along the PVA chains, through the formation of ester bonds, was confirmed by the appearance of the band centred at 1720 cm^−1^ ascribable to the formation of the ester bond between the activated carboxylic group of ferulic acid and the PVA hydroxyl groups. Moreover, the appearance of the aromatic C=C stretching band at 1604 cm^−1^ as well as the band at 1275 cm^−1^ (carboxylic acid C–O stretching) confirmed the presence of ferulic acid moiety. Finally, a significant difference in intensity of the 1142 cm^−1^ band (marked with * in PVA spectrum, Fig. 1B) was found between PVA-FA copolymer and native PVA. Any treatment upon PVA leads to a reduction of its crystallinity, resulting in a corresponding decrease in the intensity of the 1142 cm^−1^ band, as already observed on other PVA-based hydrogels [28,31,32]. The structural analysis of PVA-FA derivatives was studied also by ^1^H NMR spectroscopy in DMSO‑*d*_6_ owing to the good solubility of all the PVA-FA samples in this organic polar solvent. The ^1^H NMR analysis of all the graft copolymers confirmed the successful coupling between PVA and FA. In fact, along with the signals attributed to PVA in the up-field region, the spectra showed the presence of signals attributed to the FA residues in the down-field region. To confirm the assignment of the signal around 6.30 ppm samples were spiked with free FA. As expected, an increase in the amount of free ferulic acid corresponds to the increase of the signal attributed to the proton in beta position of the acryloyl moiety of free FA (marked with #, Fig. 2). The difference in chemical shift from the signal of FA bound to the polymer backbone (marked with *, Fig. 2) confirmed the assumption that ferulate residues are linked through ester bonds to the PVA backbone.

To estimate the grafting degree of PVA-FA derivatives, the down-field signals attributed to ferulate were integrated and the integral values were compared with that of the methylene group of PVA. All data were summarized in Table 2.

**Table 2:** Reaction ratios, grafting degree and conversion percentage PVA-FA graft copolymers copolymer.

Copolymer	AA^(a)^	AA/PVA Ratio (%)	Grafting degree (%)	Convers.^(b)^ (%)
PVA-FA1-2.5	1	2.5	1.5	60
PVA-FA1-5	1	5	4	80
PVA-FA2-3	2	3	0.5	17
PVA-FA2-6	2	6	2.2	37

(a) AA: acylating agent. (b) The conversion into ferulate was calculated from the substitution degree and stoichiometric ratio AA/PVA.

Data summarized in Table 2 confirmed that both N-acylimidazole derivatives AA1 and AA2 were able of acylating PVA, but the latter appeared less effective than the former. Overall, the grafting degree was controlled by AA/PVA stoichiometric ratio, a factor that also affected the effective conversion of the acylating agents into ferulate. Specifically, a lower AA1/PVA ratio (i.e., 2.5 %) resulted in lower grafting degree values (i.e., 1.5 %) but relatively higher conversion values (i.e., 60 %), whereas higher AA1/PVA ratio (i.e., 5 %) yielded greater grafting degree values (i.e., 4 %) and higher conversion values (i.e., 80 %). Similar results were obtained with bis-imidazolide derivative 2, although the grafting degree values and the corresponding conversion values were affected by the lower efficacy of AA2 in the acylation reaction. Interestingly, at the lowest AA2/PVA ratio (i.e., 3 %), the corresponding conversion value was around 20 %, while it became around 40 % when AA2/PVA ratio was doubled (i.e., 6 %).

### 3.2 Characterization of PVA-FA derivatives

#### 3.2.1 Solubility test

The solubility of PVA-FA derivatives was tested in three different solvents: 1) DMSO; 2) water; 3) a water solution of sodium bicarbonate (0.1 % p/p) and the results are reported in Table 3.

**Table 3:** Grafting degree (G.D. %) and solubility features of PVA-FA graft copolymers.

Copolymer	G. D. (%)	DMSO	Water	Water-Sodium Carbonate
PVA-FA1-2.5	1.5	colourless solution	colourless compact gel	yellow compact gel
PVA-FA1-5	4	colourless solution	colourless compact gel	yellow compact gel
PVA-FA2-3	0.5	colourless solution	colourless gel	yellow gel-yellow sol
PVA-FA2-6	2.2	colourless solution	colourless compact gel	yellow compact gel

All the PVA-FA samples were soluble in DMSO, while their interaction with water led to hydrogels showing different compactness depending on the grafting degree value. Samples with high grafting degree (i. e. PVA-FA1-5, PVA-FA1-2.5, and PVA-FA2-6) formed compact hydrogels, whereas PVA-FA2 and 3 hydrogels, with a grafting degree value less than 1 %, showed low compactness. When the PVA-FA samples were exposed with water containing sodium carbonate (1.0 mg/mL), the formation of yellow hydrogels occurred, showing different compactness degrees into yellow solutions. These results can be explained by assuming that the basic environment could deprotonate the phenol hydroxyl of ferulate residues, thus showing a red shift of the absorption within the visible region, resulting in the appearance of a yellow colour.

#### 3.2.2 Swelling ratio

**Fig. 3:**
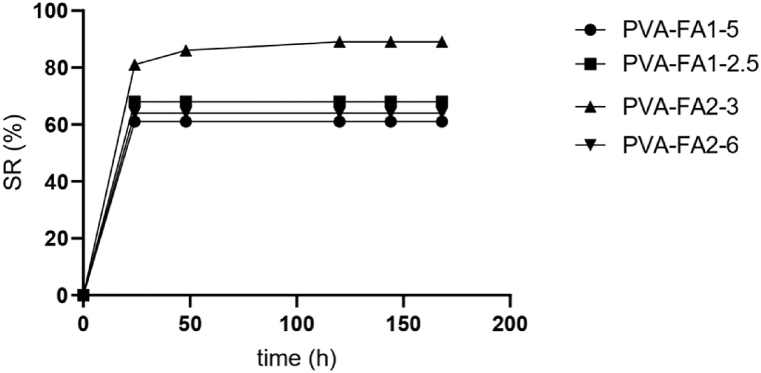
Swelling kinetics of PVA-FA hydrogels.

The swelling capacity of PVA-FA hydrogels was quantified. All samples reached the swelling equilibrium within the initial 24 h, with a swelling ratio inversely proportional to the grafting degree. The higher the grafting degree, the lower the swelling degree, consistent with a higher crosslinking density (Fig. 3).

Swelling kinetics highlighted the stability of hydrogels. No dissolution phenomena were observed after 168 h, providing evidence for the formation of stable hydrogels.

#### 3.2.3 Thermogravimetric analysis

Thermal analyses in terms of TGA and DSC were conducted to assess water-polymer interaction.

**Fig. 4:**
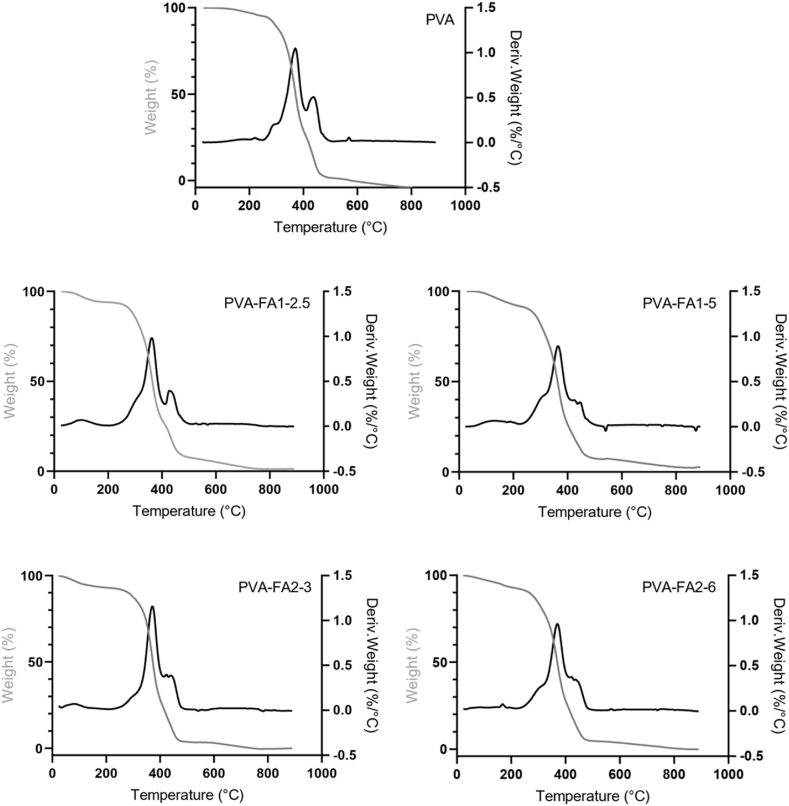
Thermograms of PVA polymer and PVA-FA hydrogels obtained plotting weight (%) and weight derivative (%/°C) versus temperature.

Thermograms of samples are reported in Fig. 4. Three main regions of weight loss can be observed: the first region, that ranges from 30 °C to 200 °C, is related to the evaporation of hydration water. The second region, that ranges from 200 °C to 400 °C, can be ascribed to the degradation of free chains. Finally, the third region (400–600 °C) is related to the degradation of condensed chains.

*Per cent* weight loss in each temperature range is reported in Table 4.

**Table 4:** Per cent weight loss as a function of temperature. R is the ratio between the weight loss in 400–600 °C range and the weight loss in 200–400 °C range. Data were reported as per cent mean value % ± SD (n = 3).

Copolymer	30–200 °C	200–400 °C	400–600 °C	R	Tot
PVA	2.9 ± 0.3 %^a^	68 ± 1 %^a^	30 ± 1%^a^	0.44^a^	100^a^
PVA-FA1-2.5	6.0 ± 0.3 %^c^	65 ± 1 %^a^	24 ± 2 %^b^	0.37^c^	99^a^
PVA-FA1-5	7.4 ± 0.3 %^b^	66 ± 1 %^a^	20 ± 1%^a^	0.30 ^b^	97 ^b^
PVA-FA2-3	6.7 ± 0.4 %^b^	65 ± 1 %^a^	25 ± 1 %^b^	0.38^c^	100^a^
PVA-FA2-6	6.8 ± 0.3 %^b^	65 ± 1 %^a^	24 ± 2%^b^	0.37^c^	100^a^

Different letters in the same column indicate significant differences (p < 0.05; ANOVA one-way, post-hoc Tukey's test).

Data indicates that the presence of FA residues along the polymer chains mainly affected its water affinity, significantly increasing the percentage of water bound to the matrix (first range). No significant difference was observed increasing the grafting degree. Interestingly, a sort of threshold can be observed. Indeed, in the presence of a high degree of grafting (i.e. PVA-FA1-5, PVA-FA1-2.5 and PVA-FA2-6), the main weight loss was recorded at about 364 °C whereas when the grafting degree is low (i.e. PVA-FA2-3) no temperature shift was observed with respect to native PVA (371 °C). In accordance with the temperature shift, a significant reduction in the polymer's stiffness was observed, as expressed by the R value, which represents the ratio between the weight losses in the 400–600 °C and in the 200–400 °C regions. For the pristine PVA R was approximately 0.44. However, in all the samples this value exhibited a significant decrease in the range of 0.30–0.38.

#### 3.2.4 Differential scanning calorimetry

**Fig. 5:**
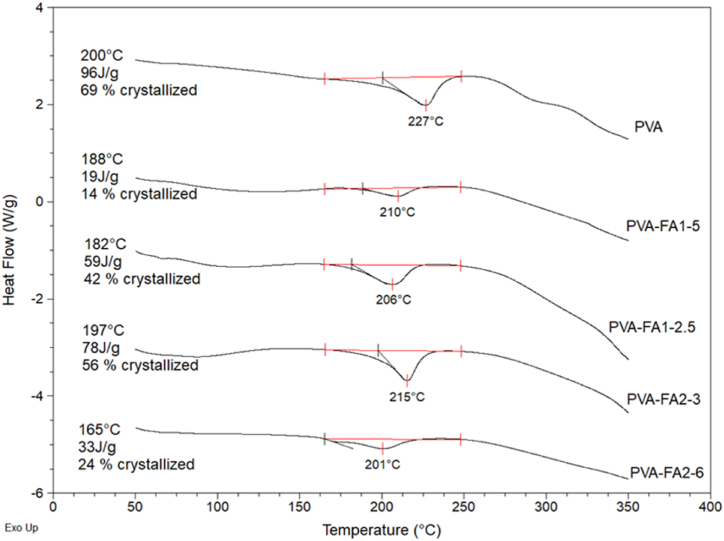
DSC thermographs melting profiles of PVA-FA derivatives.

The effect of FA moieties on the crystallinity degree of PVA was measured by DSC. Infrared analysis revealed that the FA grafting along the PVA chains caused a significant decrease of polymer crystallinity. DSC allowed to quantitatively determine the percentage of crystallinity. The heat required to melt 100 % crystalline PVA is 138.6 J/g [33]. The comparison of the thermographs of all the samples, reported in Fig. 5, highlighted that all the matrices showed a lower degree of crystallinity with respect to the native polymer due to the breakdown of the pre-established chains order, thus confirming the higher hydrophilicity of PVA derivatives as indicated also by TG analysis. Indeed, a reduction in the crystallinity of a polymer chain enhances its mobility and promote greater water interaction.

The overall hydration of olymeric hydrogel is attributed to the existence of two primary water regimes, freezing water, and non-freezing water. The first type is mainly due to free water, or adsorbed bulk water, whereas the second type can be mainly ascribed to the strictly bound water, or the hydration shell constituted by water molecules bound to polymeric chains. Freezing and non-freezing water were quantified by TGA-DSC, following a previously reported procedure [14]. In particular, the total water was quantified by TGA whereas the freezing water was obtained by DSC (Fig. S1), The non-freezing water is then calculated by difference. All results are summarized in Table 5.

**Table 5:** WH% (% total water content by TGA), Wf (freezing Water by DSC), Wnf (not freezing Water), and the corresponding percentage. Data were reported as percent mean value ± SD (n = 3).

Copolymer	WH	Wf (%)	Wnf (mg)
PVA-FA1-2.5	75 ± 2%^c^	89 ± 2 %^b^	11 ± 2%^b^
PVA-FA1-5	57 ± 3%^a^	80 ± 3 %^a^	20 ± 3%^a^
PVA-FA2-3	83 ± 1%^d^	85 ± 3 %^a,b^	15 ± 3 %^a,b^
PVA-FA2-6	66 ± 2%^b^	86 ± 3%^a,b^	14 ± 3 %^a,b^

Different letters in the same column indicate significant differences (p < 0.05; ANOVA one-way, post-hoc Tukey's test).

Despite the significant difference in terms of total water, as highlighted by both SR% (Fig. 3) and TGA measurements, all the hydrogels exhibited similar proportions of freezing and non-freezing water. The relative high percentage of non-freezing water ensures the maintenance of a sufficiently moist environment, as confirmed by the MVTR quantification reported in the following paragraph.

#### 3.2.5 Moisture vapour transmission rate (MVTR)

MVTR for PVA-FA hydrogels, native PVA (100 %) and positive control (uncovered centrifuge tube) were measured at hourly intervals for a duration of 8 h and subsequently after 24 h. A MVTR value in the range 2500–3000 g/m^2^/day guarantees sufficient moisture level without any risk of wound dehydration [34]. All PVA-FA based hydrogels showed good MVTR values within the optimal range. In accordance with thermal analyses and swelling ratio results, the highest MVTR value was found for PVA-FA2-3 (i.e., 2865 g/m^2^/day) whereas PVA-FA1-5 showed the lowest value (2512 g/m^2^/day) a condition that may potentially lead to wound dehydration. Native PVA showed a value of 3650 g/m^2^/day, close to what found by Karthick et al. [2] (3425 g/m^2^/day). Both values resulted too high, thus increasing the risk of infection at the wound site.

#### 3.2.6 Rheological analysis

**Fig. 6:**
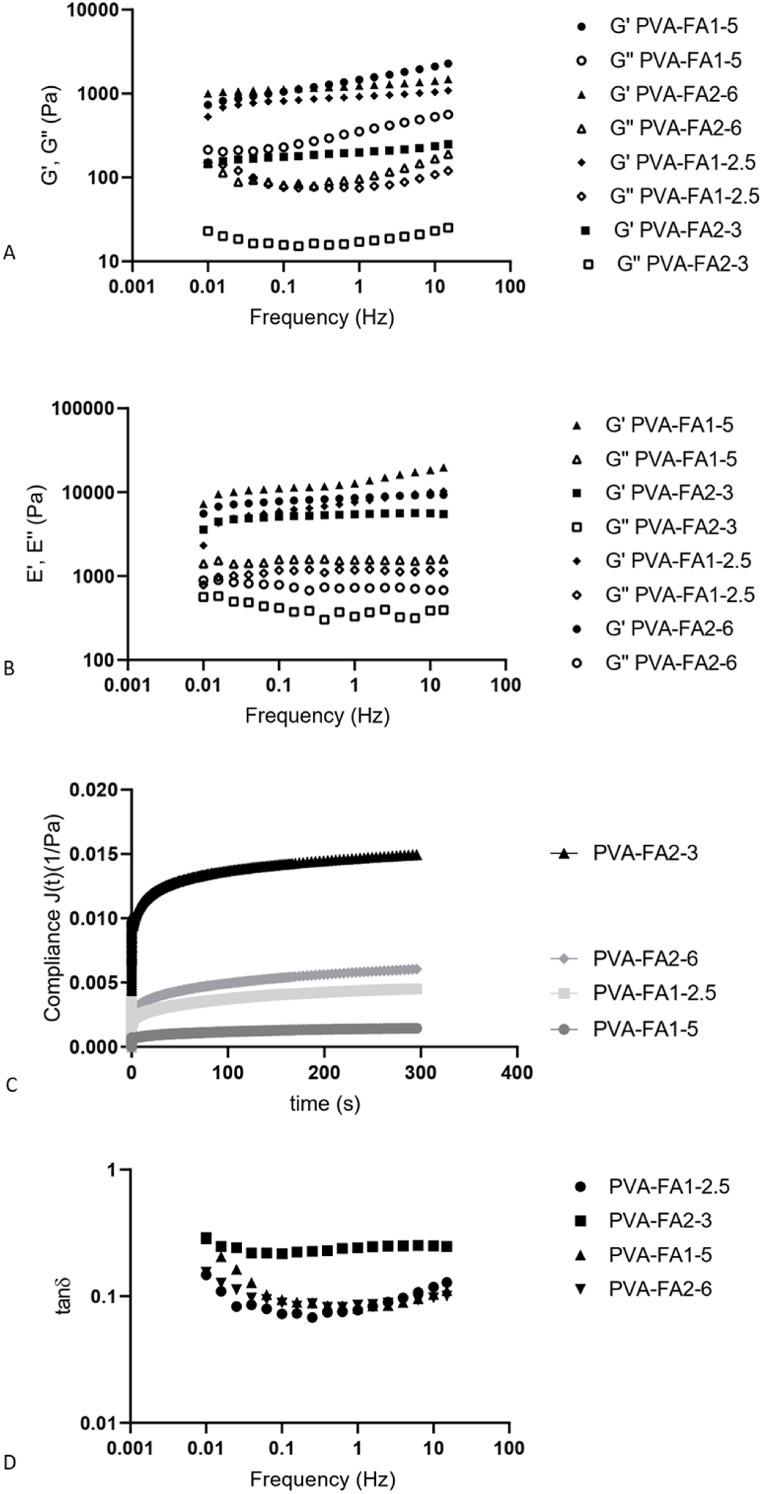
A: Gꞌ and Gꞌꞌ trends as a function of frequency; B: Eꞌ and Eꞌꞌ trends as a function of frequency; C: compliance trends; D: tan delta trends as a function of frequency.

Complete rheological tests were performed to check the behaviour of hydrogels. During the wound healing process, hydrogel dressings are inevitably exposed to external forces. To safeguard against potential harm to the wound tissue resulting from these external forces, it is essential for hydrogel-based dressings to possess appropriate mechanical properties, as emphasized in Ref. [35]. A frequency sweep test was carried out for all the samples to quantify elastic and viscous moduli as a function of angular frequency, both in shear and compression mode. Mechanical properties are depicted in Fig. 6. All the hydrogels showed G′ value greater than G″ value at all the analyzed frequencies, according to the typical “gel-like” behaviour. Moreover, they can be classified as strong gels since the difference between the two modules is about one order of magnitude. Accordingly, the phase shift, denoting the angular displacement between the oscillatory strain and stress in the examined materials measured in degrees, is notably well below the 45° threshold. (Fig. S2), so the materials showed prevalently an elastic behaviour rather than a viscous one, typical for crosslinked systems. Indeed, as reported by Clark and Murphy, a fully developed gel or “strong gel” has the property G' > G″, where both moduli (especially G′) have the further property of being nearly independent of frequency over a large frequency range [36]. The absence of a crossover-point along the entire analyzed frequency range demonstrated that the materials retained their solid like structure, both under shear and compression solicitations (Fig. 6A and B). Storage modulus G′ was also used to calculate the mesh size (ξ), defined as the distance between valid crosslinking points, basing on Rubber Elasticity Theory (RET) [37]. As mesh size increases, crosslinking densisty decreases. The crosslinking density of the produced hydrogels was determined using the methodology reported in Ref. [32] revealing a clear correlation with the grafting degree. Specifically, a higher grafting degree corresponds to an elevated elastic modulus, signifying that an increased grafting degree resulted in a greater crosslinking density, expressed as mesh size. No significant difference was observed among samples with a grafting degree higher than 1 %, i.e. PVA-FA1-5, PVA-FA1-2.5 and PVA-FA2-6, both in terms of mesh size, being 13, 15 and 14 nm, respectively, and of elastic and viscous moduli. Conversely, in the case of sample PVA-FA2-3, characterized by a grafting degree lower than 1 %, a mesh size twice as large (26 nm) and significantly lower moduli values were found.

The ability of PVA-FA based hydrogels to relax after the application of instantaneous deformation was analyzed by stress-relaxation test. Four deformation percentages were tested (5 %, 20 %, 30 % and 100 %). Regardless the applied strain all samples completely relaxed (99 %) within the first 0.1 s (Fig. S3) without undergoing a rupture, thus highlighting an optimal resistance to instantaneous deformation. The creep-recovery behaviour was also studied. It permits to determine the physical strength of a hydrogel in terms of elasticity. Compliance values are related to the internal material organization. Indeed, the presence of more extensive interactions and a diminished number of free functional groups in the polymer hydrogel matrix requires more energy to undergo deformation. Consequently, this results in a lower value for creep compliance under the applied stress [38]. All the hydrogel retracted the acquired strain quickly: this indicates that hydrogels had a high elasticity. As observed in stress-relaxation analysis, all samples showed a similar behaviour, even if the significantly lower compliance of sample PVA-FA1-5 (Fig. 6C) indicated a lower deformation for it, accordingly with its higher crosslinking density.

Numerous studies demonstrated a good correlation between shearing rheology and adhesivity in terms of tack and peel adhesion of materials. It has been reported that low frequency storage modulus Gꞌ (0.1 rad/s) correlates with tack, and high frequency Gꞌ (100 rad/s) correlates with peel [30]. Generally, low Gꞌ hydrogels have higher tack. Good correlations between Gꞌ to the cohesive strength were also reported, while the Gꞌꞌ appears to be proportional to adhesive strength. Tack and peel should be balanced for good adhesiveness. Analysing both Gꞌ and Gꞌꞌ trends, a balanced tack and peel forces for samples PVAFA1-5 and PVAFA2-3 can be observed, while there is an irregular pattern for PVAFA1-2.5 and PVAFA2-6 at the higher frequency range. In this study, PVAFA2-3 showed the highest adhesive behaviour due to its low Gꞌ value compared to the others. Adhesiveness can also be quantified and graphically represented in terms of the damping factor (tanδ or loss tangent), This property is intrinsic to materials and can be determined by calculating the ratio of Gꞌꞌ to Gꞌ. All samples showed tanδ values lower than one, thus confirming the elastic nature of hydrogels (Fig. 6D). In general, a low tanδ value is indicative of the cohesive strength, whereas high tanδ values are associated with increased adhesive strength [30]. Consequently, it can be observed that sample PVAFA2-3 not only exhibits the higher tanδ value but also maintains its frequency independence in shear mode (Fig. 6D).

#### 3.2.7 Adhesion strength

**Fig. 7:**
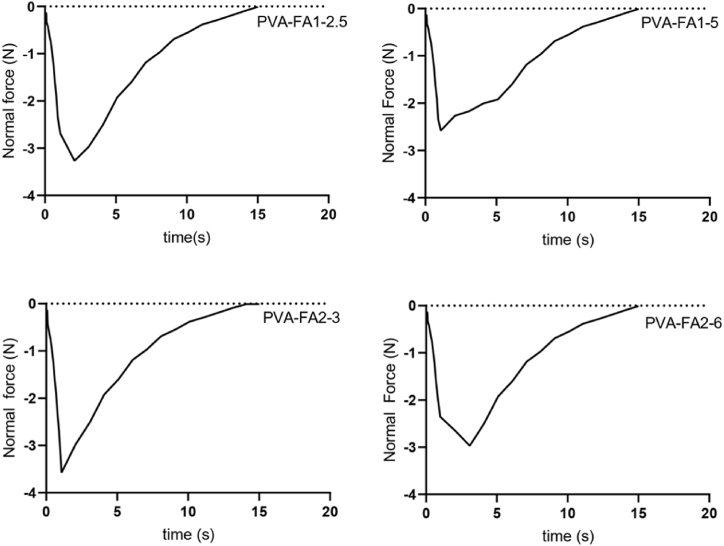
Adhesive behaviour of PVA-FA hydrogels.

The adhesive property of the PVA-FAs hydrogels was found by peel and tack test as reported by Baktwara et al. [30]. This analysis provides the strength of the bond between two surfaces (tack) and the force required to break the bond between the adhesive material and the surface (peel). The adhesion strength of all the samples was quantified by plotting peak normal force (N) against time (s). The normal force values are negative since the sample pulls the upper plate thanks to its adhesive strength and the normal force decays at zero once at the failure point. The area under the curve gives the adhesion strength. Adhesive strength is determined by calculating the ratio of the peak normal force applied to the surface area of the analyzed samples (Fig. 7). All data are summarized in Table 6.

**Table 6:** Peak normal force and adhesion strength for PVA-FA hydrogels.

Sample	Peak Normal Force (N)	Surface Area (mm^2^)	Adhesion Strength (kPa)
PVA-FA1-2.5	−3.19 ± 0.08	0.49 ± 0.01	6.51 ± 0.05
PVA-FA1-5	−2.57 ± 0.04	0.48 ± 0.03	5.36 ± 0.09
PVA-FA2-3	−3.57 ± 0.05	0.48 ± 0.02	7.45 ± 0.09
PVA-FA2-6	−2.96 ± 0.06	0.52 ± 0.03	5.70 ± 0.06

The adhesion strength for PVA-FA1-5, PVA-FA1-2.5, PVA-FA2-6 and PVA-FA2-3 was 5.36 kPa, 6.51 kPa, 5.70 kPa and 7.45 kPa, respectively. The values obtained closely align with those reported by Chen et al. in their study [39] (3.4–9.3 kPa) on self-healing, adhesive and antibacterial hydrogels based on gelatine, methacrylate and adenine acrylate. This correspondence confirms the strong adhesive properties exhibited by these series of hydrogels. Given that a higher normal force reflects an increased capacity of the hydrogel to adhere to other surfaces, it is evident that the PVA-FA2-3 hydrogel emerges as the most promising candidate for the development of wound dressings.

## 4 Conclusions

In summary, we fabricated stretchable and highly adhesive hydrogels functionalizing PVA through the insertion of ferulic acid (FA) moieties. The mechanical spectra of the fully hydrated samples

confirmed that PVA-FA hydrogels were crosslinked systems, as evidenced by the storage modulus exceeding the loss modulus (G' > G″). The developed grafting procedure permitted to modulate the grafting degree and consequently crosslinking density. The crosslinking density affected the hydrogel-water interaction thus modulating the capability to perform for the foreseen application. Basing on the swelling ratio, the MVTR and the rheological behaviour, the hydrogel obtained functionalizing PVA with 1-imidazolecarboxylate in a ratio of 3 % (PVA-FA2-3) appears as a suitable candidate for the realization of hydrogel wound dressing.

## Data availability

Data will be made available on request.

## CRediT authorship contribution statement

**Simone Pepi:** Writing – review & editing, Writing – original draft, Investigation, Formal analysis, Data curation. **Marco Paolino:** Investigation, Formal analysis, Data curation. **Mario Saletti:** Investigation, Formal analysis. **Jacopo Venditti:** Investigation, Formal analysis. **Luigi Talarico:** Investigation, Formal analysis. **Marco Andreassi:** Investigation, Formal analysis. **Germano Giuliani:** Investigation, Formal analysis, Data curation. **Gianfranco Caselli:** Conceptualization. **Roberto Artusi:** Investigation, Formal analysis. **Andrea Cappelli:** Writing – review & editing, Writing – original draft, Methodology, Funding acquisition, Data curation, Conceptualization. **Gemma Leone:** Writing – review & editing, Writing – original draft, Methodology, Data curation, Conceptualization. **Agnese Magnani:** Funding acquisition, Conceptualization. **Lucio Rovati:** Investigation, Formal analysis.

## Declaration of competing interest

The authors declare that they have no known competing financial interests or personal relationships that could have appeared to influence the work reported in this paper.
