# Supplementary material for: Ferulated Poly(vinyl alcohol) based hydrogels
Source: Heliyon. 2023 Nov 14;9(11):e22330. doi: 10.1016/j.heliyon.2023.e22330 (PMC10692910; doi:10.1016/j.heliyon.2023.e22330)
Supplement: Multimedia component 1 [file mmc1.docx]

**Ferulated Poly(vinyl alcohol) based hydrogels †**

Simone Pepi^a,c^, Marco Paolino^a^, Mario Saletti^a^, Jacopo Venditti^a^, Luigi Talarico^a,c^, Marco Andreassi^a^, Germano Giuliani^a^, Gianfranco Caselli^b^, Roberto Artusi^b^, Andrea Cappelli^*a^, Gemma Leone^*ac^, Agnese Magnani^ac^, Lucio Rovati^b^

a. Dipartimento di Biotecnologie, Chimica e Farmacia, Università di Siena, Italy

b. Rottapharm Biotech, Monza, Italy.

c. INSTM, Firenze, Italy


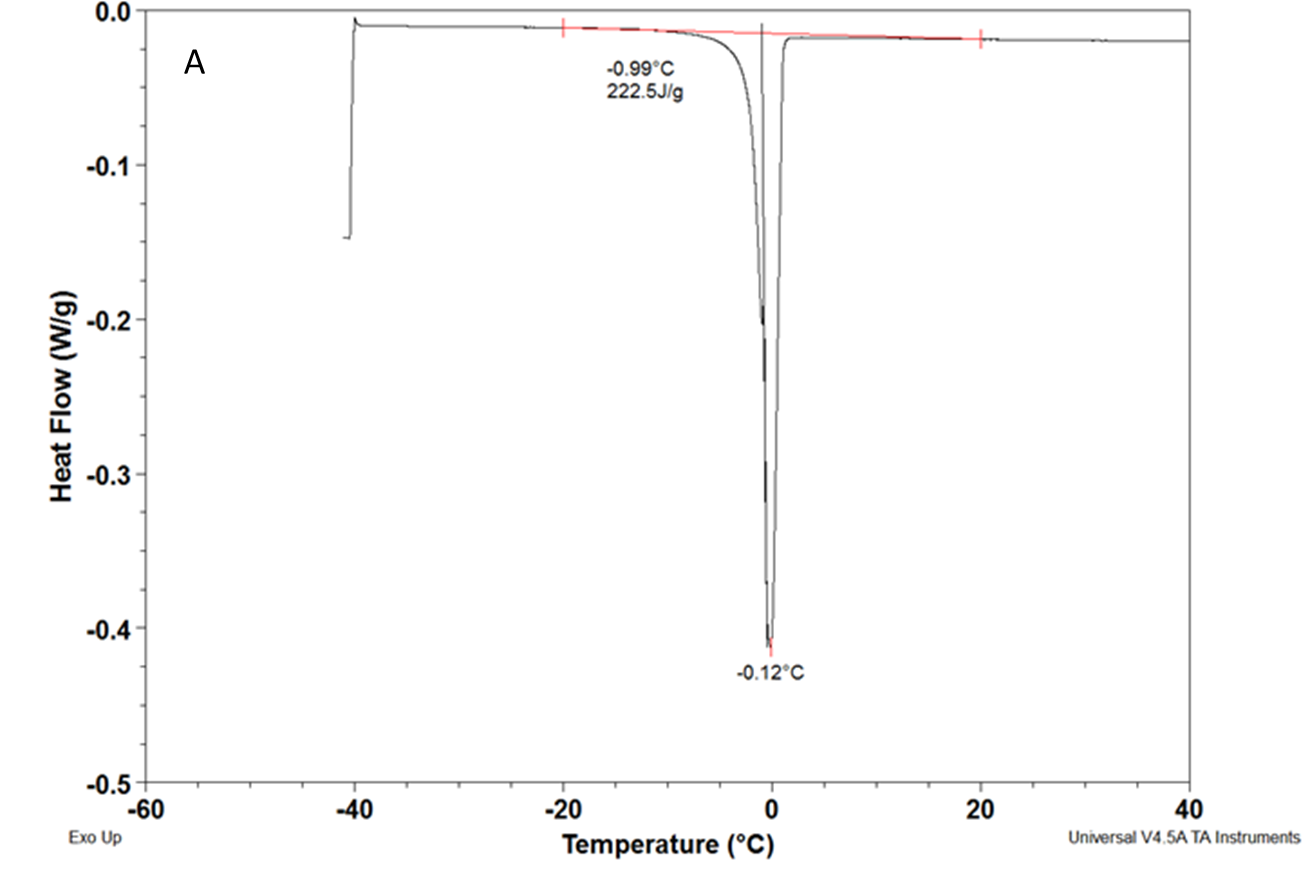


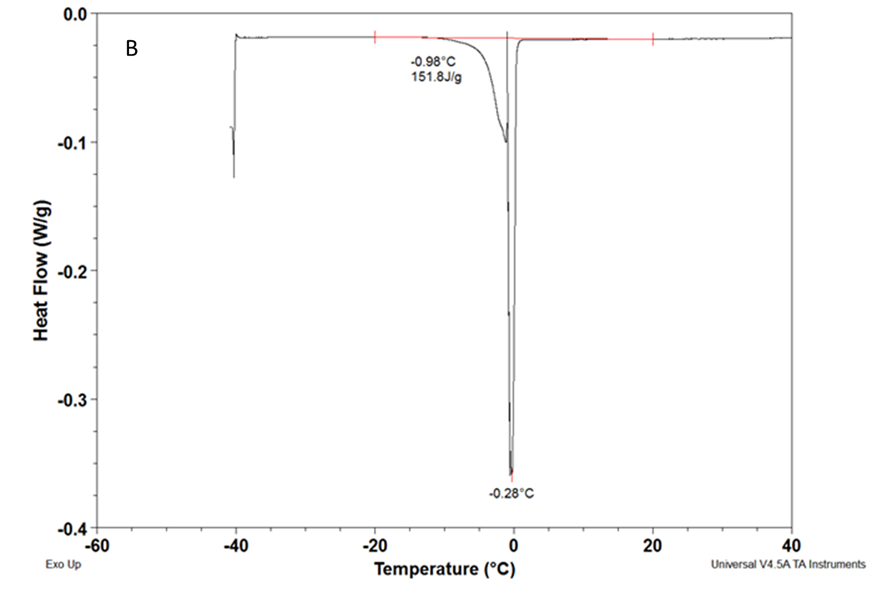


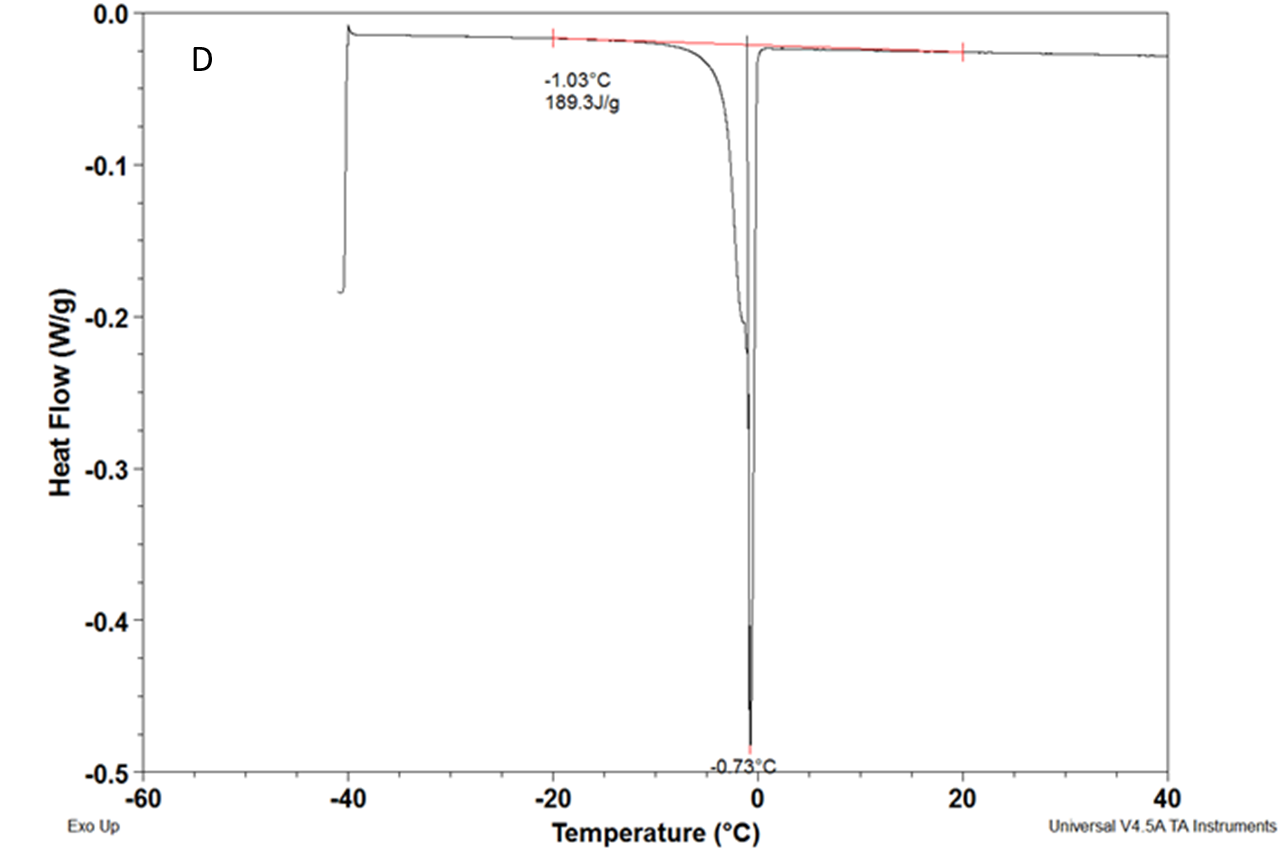


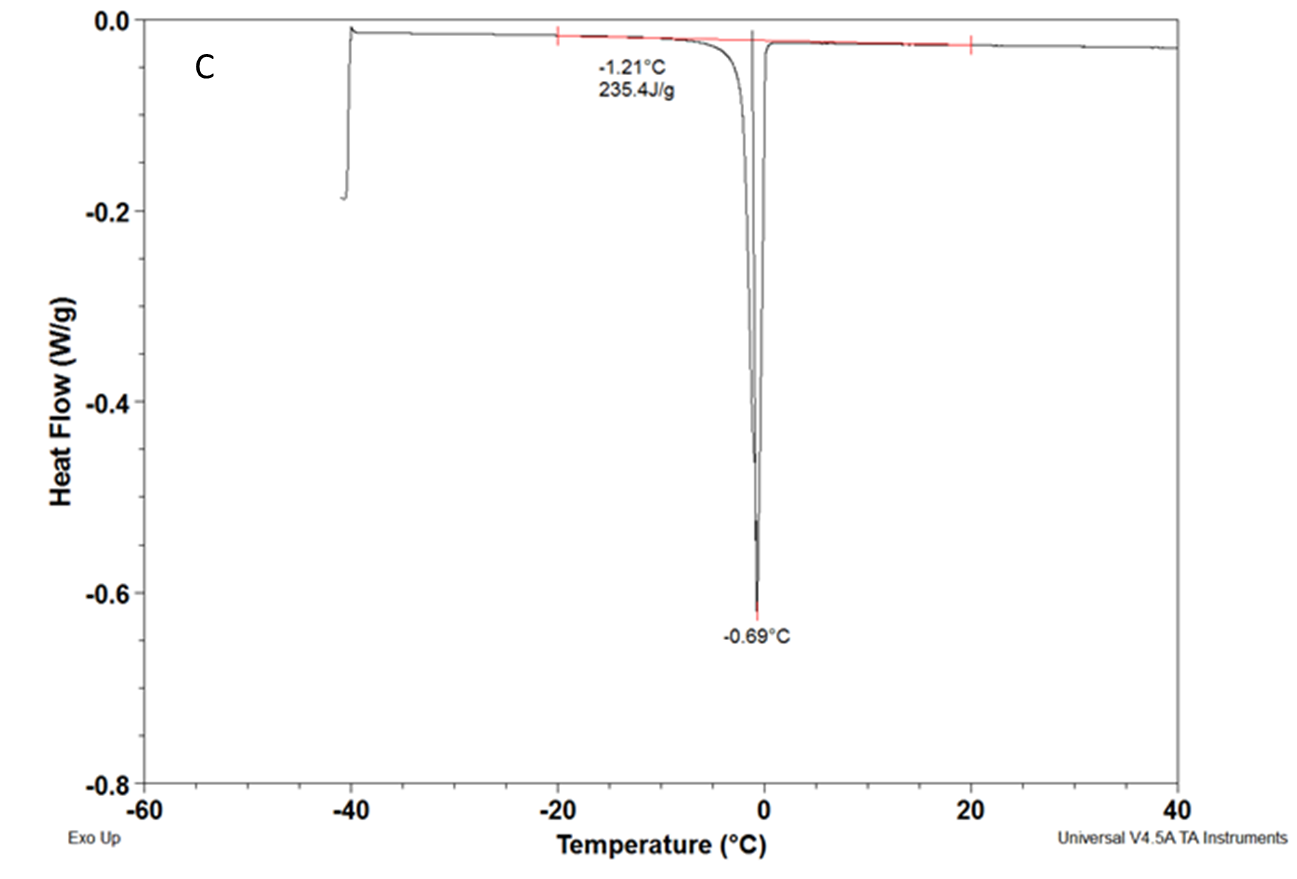


Figure S1: Ice melting thermographs of completely swollen PVA-FA based hydrogels: A: PVA-FA1-2.5; B: PVA-FA1-5; C: PVA-FA2-6; D: PVA-FA2-3


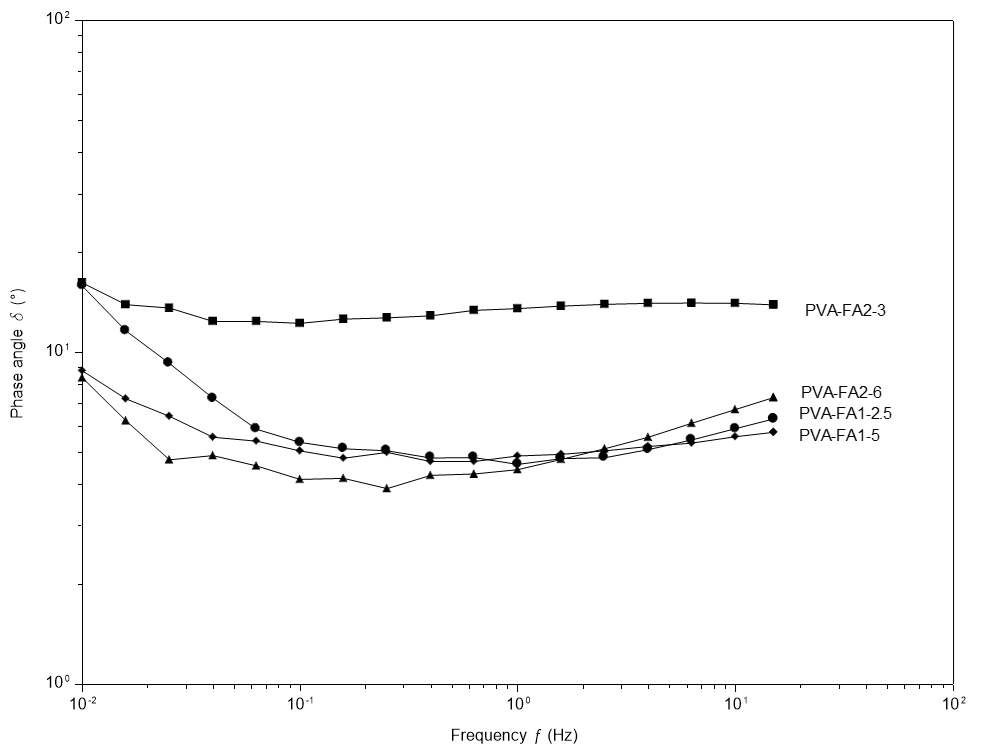


**Figure S2:** Phase angle trend of PVA-FA hydrogels as a function of increasing frequency (Hz)


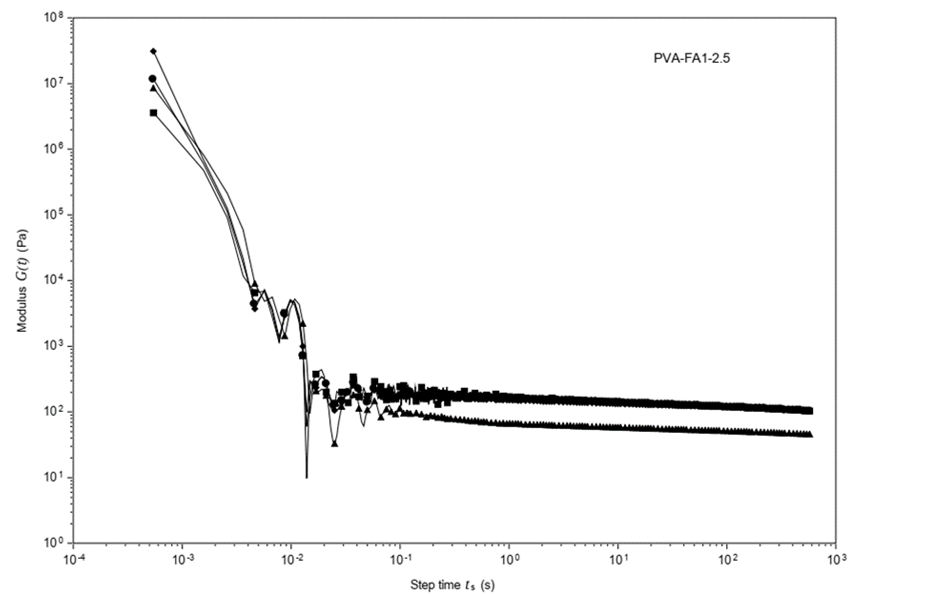


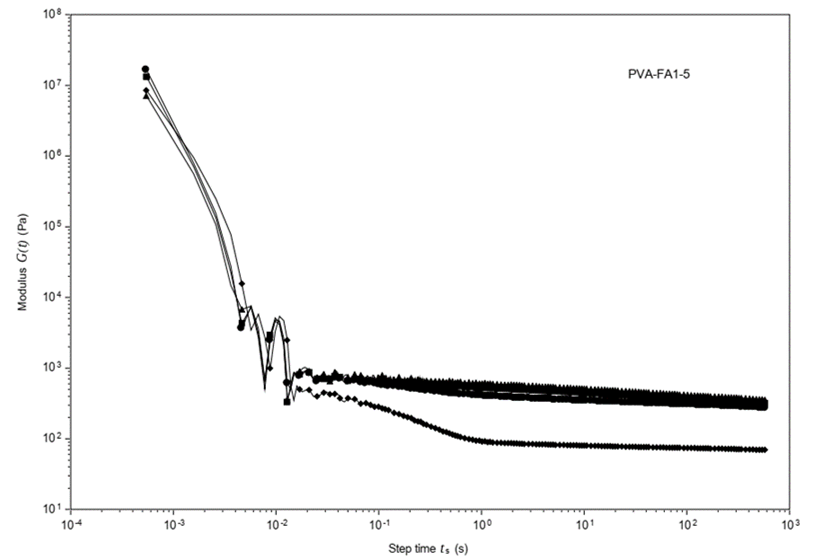


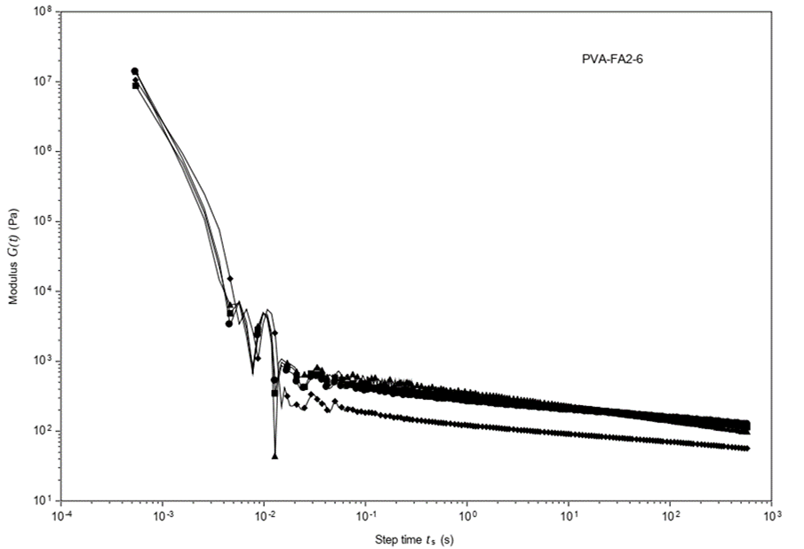


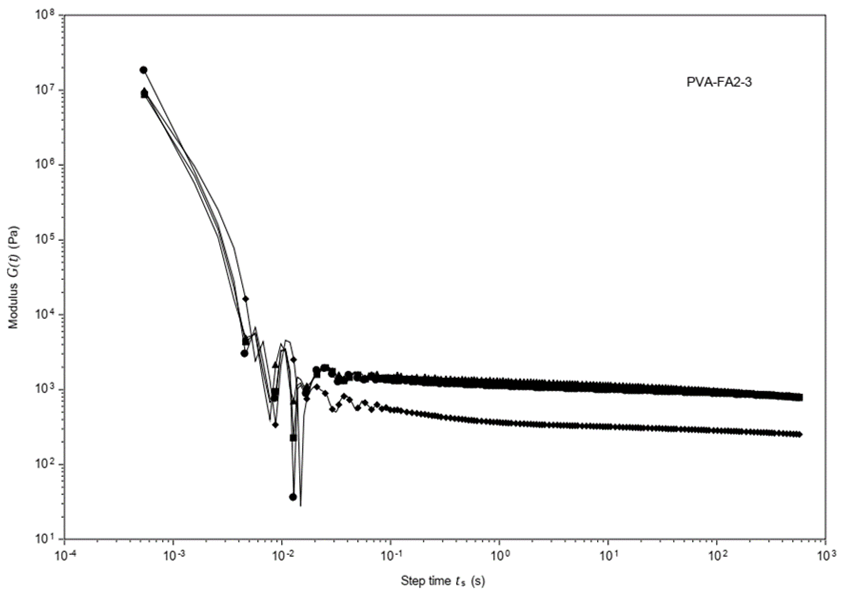


**Figure S3:** Stress-relaxation diagrams of PVA-FA hydrogels
